# Optimal two-stage spatial sampling design for estimating critical parameters of SARS-CoV-2 epidemic: Efficiency versus feasibility

**DOI:** 10.1007/s10260-023-00688-z

**Published:** 2023-03-30

**Authors:** G. Alleva, G. Arbia, P. D. Falorsi, V. Nardelli, A. Zuliani

**Affiliations:** 1 Sapienza University of Rome, Rome, Italy; 2 Catholic University of Sacred Heart, Milan, Italy; 3 University of Milan-Bicocca, Milan, Italy

**Keywords:** Anticipated variance, Local cube method, Optimal sampling design, Epidemic surveillance and monitoring, Spatial correlation

## Abstract

The COVID-19 pandemic presents an unprecedented clinical and healthcare challenge for the many medical researchers who are attempting to prevent its worldwide spread. It also presents a challenge for statisticians involved in designing appropriate sampling plans to estimate the crucial parameters of the pandemic. These plans are necessary for monitoring and surveillance of the phenomenon and evaluating health policies. In this respect, we can use spatial information and aggregate data regarding the number of verified infections (either hospitalized or in compulsory quarantine) to improve the standard two-stage sampling design broadly adopted for studying human populations. We present an optimal spatial sampling design based on spatially balanced sampling techniques. We prove its relative performance analytically in comparison to other competing sampling plans, and we also study its properties through a series of Monte Carlo experiments. Considering the optimal theoretical properties of the proposed sampling plan and its feasibility, we discuss suboptimal designs that approximate well optimality and are more readily applicable.

## Introduction

The SARS-CoV-2 pandemic has affected Western countries suddenly and in a devastating manner. The presence of many mutations of the virus after the first 2020 wave of the pandemic, together with possible future new pandemic emergencies, makes it essential to establish a system of timely monitoring and surveillance tools. The phenomenon has already been analysed in an extensive scientific literature which proposes various methods for the analysis of the COVID-19 pandemic. Italy was the first European nation to host an outbreak during the month of February 2020 (Cerqua and Stefano, 2022), so it was of relevance for the development of epidemic spread models (see Mingione et al. 2022 and Scrucca 2022 among others). Sample surveys are of paramount importance during a pandemic since they allow the estimation of the number of asymptomatic and paucisymptomatic cases. In fact, these categories of infection are not generally observed through medical swabs, which are mainly directed toward symptomatic patients (Li et al. 2020; Mizumoto et al. 2020), thus causing an underestimation of prevalence and an overestimation of the lethality rate. Ioannidis (2020) stigmatized the risk of erroneous inference based on such data.

Alleva et al. (2022) proposed an indirect sampling mechanism based on tracing the contacts of verified infected people (who could be either hospitalized or in compulsory quarantine) to build a continuous-time surveillance system to assess the prevalence of infected people in the population. The quoted methodology, although very efficient, does not consider spatial correlation among the observed data, which represent an intrinsic feature of infectious diseases (Cliff et al. 1981). Moreover, this proposal implies both innovations in the sampling design (through an indirect mechanism based on tracing) and innovation in the data collection and institutional setting. This latter innovation, unavoidable for monitoring the spread of infection, derives from the need for cooperation between different health institutions (which have the responsibility and the information on the spread of the pandemic) and the statistical agencies (which have the mandate for designing the survey and ensuring its quality). However, dealing with so many innovations could seriously jeopardise the operativity of the survey. For this reason, we need to establish a system of timely infection monitoring based on standard and well-known sampling techniques.

In the present contribution, we consider the use of two-stage sampling, a design widely used by the National Statistical Institutes to conduct surveys involving direct interviewing. The major novelty of our work is to define the factors that contribute to efficiency and to identify which components of these factors are reducible considering the feasibility of the sample design. We first define the conditions for achieving theoretical efficiency under a superpopulation model, coherent with the pandemic's evolution, which includes spatial correlation. We then examine sampling choices that, while allowing us to approach theoretical efficiency, are also practically feasible. We emphasize the role of spatial information (Grafström et al. 2012; Jauslin and Tillé, 2020) in improving a standard two-stage sampling design. Similar to past pandemics, the Covid phenomenon displays strong spatial correlation due to the mechanism through which the contagion spreads. Indeed, the disease spreads through human contact, and those who are spatially closest to infected individuals have a higher probability of contracting the disease. The early cases of epidemic are always reported in a very precise and concentrated geographic area, as it clearly shown by empirical data. As a result, spatial sampling provides lower sample variability with the same number of individuals when looking for disease-positive individuals. This feature allows to minimize the consideration of the number of people located in nearby locations that display a similar development of the disease.

In particular, aiming at a strategy that is both efficient and feasible, we consider spatially balanced sampling in the first stage (Tillé 2020) and simple random sampling in the second stage. Since infectious phenomena are positively correlated, spatial sampling allows gaining efficiency by spreading the first-stage sample over space. Moreover, the balancing strategy leverages aggregated data on the number of verified infected people: a set of data that in many countries is openly available for specific uses. Simple random sampling in the second stage does not require information at the individual unit level and ensures feasibility. To verify the effectiveness of the proposed strategy, we simulate the development of the pandemic by considering a scheme in which people are free to move around the territory and, according to their social network, can meet other people and possibly become infected. In this simulation scheme, generated by random mechanisms, the probability of getting infected depends on the individuals’ characteristics including their social network and propensity to move. To produce a realistic picture, in our simulations, we also consider the different phases of infection spreading: initial outbreak, peak and lockdown.

The rest of the paper is organized as follows. Section 2 illustrates the sampling framework. Section 3 introduces a superpopulation model that considers a positive distance-decreasing spatial correlation of the state of infection. Under our model, we can then obtain the anticipated variance (AV) of the Horvitz Thompson estimator (see Isaki and Fuller 1982; Nedyalkova and Tillé, 2008). Section 4 defines the theoretical conditions to obtain the maximum efficiency of the sampling plan and discusses its feasibility. Section 5 illustrates a model to simulate the geographical spread of the pandemic and examines the properties of the proposed method through a Monte Carlo study. Conclusions and future challenges are highlighted in Sect. 6.

## The basic sampling framework

Let us consider a target population $U$ composed of $N$ people that can refer to the inhabitants of a country or of a specific district. Let us further suppose that $U$ can be partitioned into $M$ subpopulations (called *clusters* or subareas) denoted as $U_{1} , \ldots ,U_{i} , \ldots ,U_{M} .$ According to the notation used by Sarndal et al. (1992, p. 116), the set of all clusters is indicated with the symbol $U_{{\text{I}}} = \left\{ {U_{1} , \ldots ,U_{i} , \ldots ,U_{M} } \right\}$. Each cluster is composed of $N_{i}$ individuals, with $N = \mathop \sum \nolimits_{i = 1}^{M} N_{i} .$ Furthermore, let $v_{ij} \left( {i = 1, \ldots , M; j = 1, \ldots ,N_{i} } \right)$ be the value of the target variable $v$ referring to the verified status of infection for person $j$ belonging to cluster $U_{i}$*,* with $v_{ij} = 1$ if the $ij$ individual has a verified state of infection (either hospitalized or restricted in compulsory quarantine), and $v_{ij} = 0$ otherwise. Let us further define the following quantities:${\mathcal{V}}_{i} = \mathop \sum \nolimits_{j = 1}^{{N_{i} }} v_{ij} \;{\text{and}}\;{\mathcal{V}} = \mathop \sum \nolimits_{i = 1}^{M} {\mathcal{V}}_{i}$, which represent the known totals of the verified infected people in each cluster $U_{i}$ and, respectively, in the whole population $U$. Public health authorities have full knowledge of the aggregate quantities ${\mathcal{V}}_{i}$, and they often disseminate them as open data and share them on official websites. Let $y_{ij}$ be the single observation of a dichotomous variable $y$ on the presence of infection for individual $j$ in cluster $U_{i}$, with $y_{ij}$ = 1 if the person is infected and 0 otherwise. If $v_{ij} = 1$, then we have $y_{ij} = 1$. Conversely, if $v_{ij} = 0$, it is still possible that either $y_{ij} = 1$ (that is, an infected person whose infection has not yet been verified) or $y_{ij} = 0$ (a noninfected person). Our target parameter $Y$ is then represented by the total number of infected people, that is, $Y = \mathop \sum \limits_{{i \in U_{{\text{I}}} }} \mathop \sum \limits_{{j \in U_{i} }} y_{ij} = \mathop \sum \limits_{{i \in U_{{\text{I}}} }} Y_{i} ,$ where $Y_{i}$ indicates the number of infected people in the $i - th$ cluster. In this informative context, the known values of the number of verified infected people (${\mathcal{V}}_{i}$) represent an auxiliary variable available for estimating the target parameter $Y$ (the true number of infected people).

Our proposed sample design can be illustrated as follows.

First, we select a sample *S* from *U* using a two-stage random sampling design without replacement. According to the specific sampling context, the primary sample units (PSUs) may correspond to different levels of aggregations, e.g., municipalities or census enumeration areas.

The sampling process starts with drawing a first-stage sample of clusters, $S_{{\text{I}}}$, of fixed size $m$. $S_{{\text{I}}}$ is selected without replacement from $U_{{\text{I}}} ,$ with inclusion probabilities $\pi_{{{\text{I}}i}} \left( {i = 1,2, \ldots ,M} \right)$. A standard solution in two-stage random sampling designs is to select cluster $i$ with probability proportional to size (PPS), that is $\pi_{{{\text{I}}i}} = m\frac{{N_{i} }}{N}.$

A second-stage sample, say $S_{{{\text{II}}i}}$, of fixed size $\overline{n}$ is drawn from each cluster $U_{i}$ selected in the first stage by drawing the units with a simple random sample without-replacement (SRSWOR for short) design. The second-stage inclusion probability of people in the sampled cluster $i$, say $\pi_{{{\text{II}}i}}$, is then given by $\pi_{{{\text{II}}i}} = \frac{{\overline{n}}}{{N_{i} }}.$

As a consequence, the final inclusion probability of person $j$ being selected from cluster $i$ is given by $\pi_{ij} = \pi_{{{\text{I}}i}} \pi_{{{\text{II}}i}} = m\frac{{N_{i} }}{N}\frac{{\overline{n}}}{{N_{i} }} = m\frac{{\overline{n}}}{N}.$

The sampling is *self-weighting* (Murthy and Sethi 1965) in the sense that all the units in $U$ have an equal probability of being selected irrespective of their cluster. The *self-weighting* property defines a sampling design that simplifies the data analysis phase because it avoids the complications resulting from variable weights.

Even if the first-stage sampling is based on the same vector of first-order inclusion probabilities $\pi_{{{\text{I}}i}} \left( {i = 1, \ldots ,M} \right)$, the selection can be carried out using different algorithms, leading to different first-stage sampling designs in relation to two important characteristics, namely, balancing and spreading.

In the first stage, let ${\varvec{d}}_{Ii}$ be a vector of auxiliary variables available for cluster $U_{i} .$ Sample $S_{{\text{I}}}$ is said to be *balanced* on the ${\varvec{d}}_{Ii}$ variables if:

1 $$\mathop \sum \limits_{{i \in S_{I} }} \frac{{{\varvec{d}}_{Ii} }}{{\pi_{Ii} }} = \mathop \sum \limits_{{i \in U_{I} }} {\varvec{d}}_{Ii} .$$

Moreover, the sample is said to be *approximately balanced* if $\mathop \sum \limits_{{i \in U_{{\text{I}}} }} {\varvec{d}}_{{{\text{I}}i}} \;{\text{is}}\;{\text{close}}\;{\text{to}}\mathop \sum \limits_{{i \in S_{{\text{I}}} }} \frac{{{\varvec{d}}_{{{\text{I}}i}} }}{{\pi_{{{\text{I}}i}} }}.$

Deville and Tillé (2005, p. 577) showed that several customary sampling designs may be considered special cases of balanced sampling. If we define the balancing variables in Expression (1) as ${\varvec{d}}_{{{\text{I}}i}} = \pi_{{{\text{I}}i}} ,$ then the sampling selection ensures planned sample sizes $m$ for each sampling selection. Deville and Tillé (2004; p. 905) proved that a balanced sampling design always exists if we define the ${\varvec{d}}_{{{\text{I}}i}}$ variables as ${\varvec{d}}_{{{\text{I}}i}} = \pi_{{{\text{I}}i}}$ and if $m = \mathop \sum \limits_{{i \in U_{{\text{I}}} }} \pi_{{{\text{I}}i}} ,$ is an integer. Balanced samples may be drawn using the cube method (Deville and Tillé, 2004). Usually, the sample $S_{{\text{I}}}$ is only approximately balanced for generic vectors of ${\varvec{d}}_{{{\text{I}}i}}$ of real values.

The *spreading* of sample units in space is necessary to improve the efficiency of the estimators in situations characterized by positive spatial correlation of the target variable. An example of sampling designs satisfying this desirable feature is the so-called dependent unit spatial sampling technique (DUST) method (Arbia 1993), which was subsequently improved by Arbia and Switzer (1994). More recently, Grafström (2012) and Grafström et al. (2012) introduced two methods called *correlated Poisson sampling* and the *local pivotal method* (LP), respectively, which enable the selection of unequal probability samples of fixed size that are well spread over the population. Both algorithms use the distance between the population units to create low joint inclusion probabilities for nearby units, thus forcing the samples to be well dispersed. Combining the notion of balancing with that of spreading, Grafström and Tillé (2013) proposed the *local cube* (LC) method, which enables the selection of samples that are balanced on several auxiliary variables and at the same time are also well spread for some variables, which can be geographical coordinates. In this sense, the LC method can be considered an extension of the LP method. Moreover, Grafström and Lundström (2013) demonstrated that well-spread balanced samples in space are balanced on auxiliary variables even if the target parameters are nonlinear in the auxiliary variables. Indeed, suppose we have a well-spread sampling where the distance among the units is defined in terms of some auxiliary variable. In that case, we obtain a sample approximately balanced on nonlinear functions of the auxiliary variables.

Survey enumerators verify the status of infection (e.g., through a swab) on each of the $m \times \overline{n}$ people selected in the sample, thus quantifying the values of variable $y_{ij}$. The Horvitz-Thompson (HT) estimator (Horvitz and Thompson 1952) of $Y$ is then provided by.

$$\hat{Y} = \mathop \sum \limits_{{i \in S_{{\text{I}}} }} \mathop \sum \limits_{{j \in S_{{{\text{II}}i}} }} y_{ij} \frac{N}{{m\overline{n}}} = \mathop \sum \limits_{{i \in S_{{\text{I}}} }} \hat{Y}_{i} \frac{1}{{\pi_{{{\text{I}}i}} }},$$ where $$\hat{Y}_{i} = \mathop \sum \limits_{{j \in S_{{{\text{II}}i}} }} y_{ij} \frac{{N_{i} }}{{\overline{n}}}{ }.$$

## Anticipated variance

Consider the following generalized linear Model *M*:

2 $$y_{ij} = \tilde{y}_{ij} + u_{ij}$$

where $\tilde{y}_{ij} = \Pr \left( {y_{ij} = 1} \right),$ and $u_{ij}$ are random errors, $E_{M} \left( {u_{ij} } \right) = 0$, $V_{M} \left( {u_{ij} } \right) = \sigma_{u}^{2}$ and $Cov_{M} \left( {u_{ij} ,u_{\ell k} } \right) = \sigma_{u}^{2} \rho_{ij,\ell k}$. $E_{M} \left( \cdot \right)$, $V_{M} \left( \cdot \right)$ and $Cov_{M} \left( { \cdot , \cdot } \right)$ are the expectation, variance and covariance under the model, respectively, and $\sigma_{u}^{2}$ is the homoscedastic error variance. Generally, the spatial correlation parameter $\rho_{ij,\ell k}$ is assumed to be a decreasing function of the distance $\delta_{ij,\ell k}$ between unit $j$ belonging to cluster $U_{i}$ and unit $k$ belonging to cluster $U_{l}$. Grafström and Tillé (2013) proposed specifying this term as follows:

3 $$\rho_{ij,\ell k} = \rho^{{\delta_{ij,\ell k} }}$$

where $0 \le \rho \le 1$. The probability $\tilde{y}_{ij}$ can be modelled as a Lipschitz continuous function $\tilde{y}_{ij} = g\left( {{\varvec{x}}_{ij} } \right),$ where ${\varvec{x}}_{ij}$ is a column vector of auxiliary variables specific for unity $ij$. Denoting with $E_{P} \left( \cdot \right)$ the expectation over repeated sampling, the accuracy of the proposed sampling strategy may be measured by the anticipated variance $AV\left( {\hat{Y}} \right) = E_{P} E_{M} \left( {\hat{Y} - Y} \right)^{2} .$ Adding and subtracting the term $E_{P} E_{M} \left( {\hat{Y}} \right) = \tilde{Y}$ on the right-hand side of $AV$, we have $AV\left( {\hat{Y}} \right) = E_{P} E_{M} \left[ {\hat{Y} - \tilde{Y}} \right]^{2} + E_{P} E_{M} \left[ {\tilde{Y} - Y} \right]^{2} + 2E_{P} E_{M} \left[ {\left( {\hat{Y} - \tilde{Y}} \right)\left( {\tilde{Y} - Y} \right)} \right].$ From Kendall and Stuart (1976, p. 196), we have $E_{P} E_{M} \left( {\hat{Y} - \tilde{Y}} \right)^{2} = V_{P} \left[ {E_{M} \left( {\hat{Y}} \right)} \right] + E_{P} \left[ {V_{M} \left( {\hat{Y}} \right)} \right]$. Furthermore, from Alleva et al. (2022), we have $E_{P} E_{M} \left( {\tilde{Y} - Y} \right)^{2} + 2E_{P} E_{M} \left[ {\left( {\hat{Y} - \tilde{Y}} \right)\left( {\tilde{Y} - Y} \right)} \right] = - V_{M} \left( Y \right).$

Joining together the previous results, we obtain

4 $$\begin{aligned} AV\left( {\hat{Y}} \right) = & \;V_{P} \left[ {E_{M} \left( {\hat{Y}} \right)} \right] + E_{P} \left[ {V_{M} \left( {\hat{Y}} \right)} \right] - V_{M} \left( Y \right) \\ = & \;V_{P} \left( {\mathop \sum \limits_{{i \in S_{I} }} \tilde{Y}_{i} \frac{1}{{\pi_{Ii} }} - \mathop \sum \limits_{{i \in U_{I} }} \tilde{Y}_{i} } \right) + \mathop \sum \limits_{{i \in U_{I} }} \frac{1}{{\pi_{Ii} }}N_{i} \left( {\frac{{N_{i} - \overline{n}}}{{\overline{n}}}} \right)\sigma_{{II\tilde{y}_{i} }}^{2} + \\ & \; + \left\{ {\mathop \sum \limits_{{i \in U_{I} }} \frac{1}{{\pi_{Ii} }}\sigma_{u}^{2} \left[ {\left( {\frac{{N_{i}^{2} }}{{\overline{n}}}\left( {1 + \left( {\overline{n} - 1} \right) \overline{\rho }_{i} } \right)} \right) + \left( {\mathop \sum \limits_{\ell \ne i} \frac{1}{{\pi_{I\ell } }}\pi_{Ii,I\ell } N_{i} N_{\ell } \overline{\rho }_{i,\ell } } \right)} \right]} \right\} + \\ & \; - \sigma_{u}^{2} \mathop \sum \limits_{{i \in U_{I} }} N_{i} \left\{ {\left[ {1 + \left( {N_{i} - 1} \right)\overline{\rho }_{i} } \right] + \left[ {\mathop \sum \limits_{\ell \ne i} N_{\ell } \overline{\rho }_{i,\ell } } \right]} \right\} \\ \end{aligned}$$

where $\pi_{Ii,I\ell }$ is the joint inclusion probability of selecting clusters $U_{i}$ and $U_{\ell }$ in the first-stage sampling. $\tilde{Y}_{i} = \mathop \sum \limits_{{j \in U_{i} }} \tilde{y}_{ij} = \mathop \sum \limits_{{j \in U_{i} }} g\left( {{\varvec{x}}_{ij} } \right),\sigma_{{II\tilde{y}_{i} }}^{2} = \frac{1}{{N_{i} - 1}}\mathop \sum \limits_{{j \in U_{i} }} \left( {\tilde{y}_{ij} - \frac{{\tilde{Y}_{i} }}{{N_{i} }}} \right)^{2} ,$ and the spatial correlation terms are, respectively, $\overline{\rho }_{i} = \frac{1}{{N_{i} \left( {N_{i} - 1} \right)}}\mathop \sum \limits_{{j \in U_{i} }} \mathop \sum \limits_{k \ne j} \rho_{ij,ik}$ and $\overline{\rho }_{i,\ell } = \frac{1}{{N_{i} N_{\ell } }}\mathop \sum \limits_{{j \in U_{i} }} \mathop \sum \limits_{{k \in U_{\ell } }} \rho_{ij,\ell k} .$

The first component of the anticipated variance, $V_{P} \left[ {E_{M} \left( {\hat{Y}} \right)} \right]$, is given by Expression (4) and can be easily derived using Theorem 11.1 of Cochran (1977) considering that $V_{P} \left[ {E_{M} \left( {\hat{Y}} \right)} \right] = V_{P} \left[ {\mathop \sum \limits_{{i \in S_{I} }} \mathop \sum \limits_{{j \in S_{IIi} }} \tilde{y}_{ij} \frac{N}{{m\overline{n}}}} \right].$ The second component, $E_{P} \left[ {V_{M} \left( {\hat{Y}} \right)} \right]$, is given by Expression (4) and can be easily obtained from the following result:

$$E_{P} \left[ {V_{M} \left( {\hat{Y}} \right)} \right] = \mathop \sum \limits_{{i \in U_{I} }} E_{P} \left[ {V_{M} \left( {\hat{Y}_{i} \frac{N}{{N_{i} m}}\lambda_{i} } \right)} \right] + \mathop \sum \limits_{{i \in U_{I} }} \mathop \sum \limits_{\ell \ne i} E_{P} \left[ {Cov_{M} \left( {\hat{Y}_{i} \frac{1}{{\pi_{Ii} }},\hat{Y}_{\ell } \frac{1}{{\pi_{I\ell } }}\lambda_{i} \lambda_{\ell } } \right)} \right],$$

where $\lambda_{i} = 1{\text{ if }}U_{i} \in S_{{\text{I}}}$ and $\lambda_{i} = 0$ otherwise, and $\lambda_{j|i} = 1{\text{ if }}j \in S_{{{\text{II}}i}}$ and $\lambda_{j|i} = 0$ otherwise. The third component in Eq. (3), $V_{M} \left( Y \right)$, is a fixed component depending on the population characteristics and is given by Expression (4). Finally, with some straightforward manipulation of previous equations, we may express the anticipated variance of the *Horvitz–hompson* estimator under the adopted model as follows:

5 $$AV\left( {\hat{Y}} \right) = A + B + C + D + E - V_{M} \left( Y \right)$$

where

6 $$\begin{aligned} A = & \;\sigma_{u}^{2} \mathop \sum \limits_{{i \in U_{I} }} \mathop \sum \limits_{\ell \ne i} \frac{{N_{i} }}{{\pi_{Ii} }}\frac{{N_{\ell } }}{{\pi_{I\ell } }}\pi_{Ii,I\ell } \overline{\rho }_{i,\ell } { }, \\ B = & \;V_{IP} \left( {\mathop \sum \limits_{{i \in S_{I} }} \tilde{Y}_{i} \frac{1}{{\pi_{Ii} }} - \mathop \sum \limits_{{i \in U_{I} }} \tilde{Y}_{i} } \right), \\ C = & \;\mathop \sum \limits_{{i \in U_{{\text{I}}} }} \mathop \sum \limits_{{j \in U_{i} }} \mathop \sum \limits_{k \ne j} \sigma_{u}^{2} \rho_{ij,ik} \frac{1}{{\pi_{{{\text{I}}i}} }}\frac{{\pi_{{{\text{II}}ij,{\text{II}}ik}} }}{{\pi_{{{\text{II}}i}}^{2} }} = \sigma_{u}^{2} \mathop \sum \limits_{{i \in U_{{\text{I}}} }} \frac{1}{{\pi_{{{\text{I}}i}} }}\frac{{N_{i}^{2} }}{{\overline{n}}}\left( {\overline{n} - 1} \right)\overline{\rho }_{i} , \\ D = & \;\mathop \sum \limits_{{i \in U_{{\text{I}}} }} \frac{1}{{\pi_{{{\text{I}}i}} }}N_{i} \left( {\frac{{N_{i} - \overline{n}}}{{\overline{n}}}} \right)\sigma_{{{\text{II}}\tilde{y}}}^{2} ,\quad E = \mathop \sum \limits_{{i \in U_{{\text{I}}} }} \frac{1}{{\pi_{{{\text{I}}i}} }}\frac{{N_{i}^{2} }}{{\overline{n}}}\sigma_{u}^{2} , \\ \end{aligned}$$

in which $\pi_{{{\text{II}}ij,{\text{II}}ik}} = E_{P} \left( {\lambda_{j|i} \lambda_{k|i} |U_{i} \in S_{{\text{I}}} } \right)$, with $\pi_{{{\text{II}}ij,{\text{II}}ik}} = \overline{n}\left( {\overline{n} - 1} \right)/N_{i} \left( {N_{i} - 1} \right)$ in an SRSWOR design in the second stage.

## Efficiency versus feasibility

To achieve efficiency, we adopt a sampling algorithm for the first-stage sampling which ensures that joint inclusion probabilities ${\varvec{\pi}}_{{{\mathbf{I}}{\varvec{i}},{\mathbf{I}}\ell }}$ are small whenever $\varvec{\bar{\rho }}_{{\user2{i},\varvec{l}}}$ is large in order to minimize the term ${\varvec{A}}$ of 3.3. As demonstrated in Theorem 1 in Grafström and Lundström (2013), if the first-stage sampling is well spread on the totals ${\varvec{d}}_{{{\mathbf{I}}{\varvec{i}}}} = {\varvec{X}}_{{\varvec{i}}} = \mathop \sum \limits_{{{\varvec{j}} \in {\varvec{U}}_{{\varvec{i}}} }} {\varvec{x}}_{{{\varvec{ij}}}}$ and we balance on the same totals, then we balance on the theoretical unknown values $\tilde{\user2{Y}}_{{\varvec{i}}} = \mathop \sum \limits_{{{\varvec{j}} \in {\varvec{U}}_{{\varvec{i}}} }} {\varvec{g}}\left( {{\varvec{x}}_{{{\varvec{ij}}}} } \right).$ In this way, the term ${\varvec{B}}$ of Eq. (6) tends to be tiny because $\mathop \sum \limits_{{{\varvec{i}} \in {\varvec{S}}_{{\mathbf{I}}} }} \tilde{\user2{Y}}_{{\varvec{i}}} \frac{1}{{{\varvec{\pi}}_{{{\mathbf{I}}{\varvec{i}}}} }} \cong \mathop \sum \limits_{{{\varvec{i}} \in {\varvec{U}}_{{\mathbf{I}}} }} \tilde{\user2{Y}}_{{\varvec{i}}} = \tilde{\user2{Y}}.$ We may reduce the value of the term ${\varvec{C}}$ of Eq. (5) by geographically spreading the second-stage sampling into the clusters. Suppose now that we adopt an LP sampling strategy in each cluster by geographically spreading the sample. This produces the effect of having joint inclusion probabilities ${\varvec{\pi}}_{{{\mathbf{II}}{\varvec{ij}},{\mathbf{II}}{\varvec{ik}}\left( {{\varvec{LP}}} \right)}}$ that are very small when the units are close and the correlation ${\varvec{\rho}}_{{{\varvec{ij}},{\varvec{ik}}}}$ is high. Furthermore, if we geographically spread the second-stage sampling on the ${\varvec{x}}_{{{\varvec{ij}}}}$ values and balance on the same variables, we obtain the balancing on the theoretical unknown values ${\varvec{g}}\left( {{\varvec{x}}_{{{\varvec{ij}}}} } \right)$. In this way, the term ${\varvec{D}}$ in Eq. (5) tends to be negligible because, due to the balancing, we have $\mathop \sum \limits_{{{\varvec{i}} \in {\varvec{S}}_{{\mathbf{I}}} }} \tilde{\user2{y}}_{{{\varvec{ij}}}} \frac{1}{{{\varvec{\pi}}_{{{\mathbf{II}}{\varvec{i}}}} }} \cong \tilde{\user2{Y}}_{{\varvec{i}}} .$

In synthesis, taking as fixed the first-order inclusion probabilities $\pi_{{{\text{I}}i}} = mN_{i} /N$ and $\pi_{{{\text{II}}i}} = \overline{n}/N_{i}$ (for $i \in U_{{\text{I}}}$), the maximum efficiency is achieved by spreading and balancing both stages of the sampling selection. With this strategy, the term $E$ becomes the dominant term of the AV, which can be expressed as $AV\left( {{ }\hat{Y}} \right) \cong \mathop \sum \nolimits_{i = 1}^{M} \frac{1}{{\pi_{{{\text{I}}i}} }}N_{i}^{2} \frac{{\sigma_{u}^{2} }}{{\overline{n}}} - V_{M} \left( Y \right) = E - V_{M} \left( Y \right).$

Under the constraint that the first-stage sample size is fixed $\mathop \sum \nolimits_{i = 1}^{M} \pi_{{{\text{I}}i}} = m,$ and by using a Lagrangian function, we find that the minimum in $\pi_{{{\text{I}}i}}$ of $\mathop \sum \nolimits_{i = 1}^{M} \frac{1}{{\pi_{{{\text{I}}i}} }}N_{i}^{2} \frac{{\sigma_{u}^{2} }}{{\overline{n}}}$ is given by.

$$\pi_{{{\text{I}}i}} = mN_{i} \left( {\sigma_{u} /\sqrt {\overline{n}} } \right)/\mathop \sum \nolimits_{\ell = 1}^{M} N_{\ell } \left( {\sigma_{u} /\sqrt {\overline{n}} } \right) = \frac{{mN_{i} }}{N},$$ provided that $$mN_{i} \left( {\sigma_{u} /\sqrt {\overline{n}} } \right) \le \mathop \sum \nolimits_{\ell = 1}^{M} N_{\ell } \left( {\sigma_{u} /\sqrt {\overline{n}} } \right).$$

Therefore, we see that the PPS solution, given by $\pi_{{{\text{I}}i}} = m\frac{{N_{i} }}{N}$ for defining the first-stage inclusion probabilities, is the *optimal* solution when spreading and balancing the sampling in both stages.

Above, we implicitly hypothesized that balancing and scattering of the sample in each of the two stages may nullify (more or less) the terms $A$, $B$, $C$ and $D$ in Eq. (5).

However, in designing practically feasible sampling strategies, we cannot completely neglect any of these terms. All feasible designs leave a residual that we cannot eliminate. We can better study the residuals by reformulating the AV as follows:

7 $$AV\left( {\hat{Y}} \right) = \left( {A^{*} + R_{A} } \right) + \left( {B^{*} + R_{B} } \right) + \left( {C^{*} + R_{C} } \right) + \left( {D^{*} + R_{D} } \right) + E - V_{M} \left( Y \right),$$

where $A = A^{*} + R_{A}$, $B = B^{*} + R_{B}$, $C = C^{*} + R_{C}$, and $D = D^{*} + R_{D}$, with $A^{*} , B^{*}$, $C^{*}$ and $D^{*}$ representing, respectively, the elements of the components $A$, $B$, $C$ and $D$ that can be cancelled by a proper choice of the sampling designs. Conversely, the terms $R_{A} , R_{B} , R_{C}$ and $R_{D}$ represent the unavoidable components. The greater the terms $A^{*} , B^{*}$, $C^{*}$ and $D^{*}$ approach the respective components $A$, $B$, $C$ and $D$, the greater the residuals $R$ become negligible and the sampling design approaches the maximum efficiency.

Let us first consider spreading. Having the clusters' spatial coordinates, we can quickly spread the first-stage sample on the geographical variables. For all practical purposes, we can assume that the joint probabilities obtained through the LP algorithm are a good approximation of the optimal joint inclusion probabilities that minimize the term $R_{A}$. Therefore, the terms $A^{*}$ and $R_{A}$ may be approximately defined as follows:

8 $$A^{*} \cong \sigma_{u}^{2} \mathop \sum \limits_{{i \in U_{I} }} \mathop \sum \limits_{\ell \ne i} \frac{{N_{i} }}{{\pi_{Ii} }}\frac{{N_{\ell } }}{{\pi_{I\ell } }}\overline{\rho }_{i,\ell } \left( {\pi_{{Ii,I\ell \left( {FPPS} \right)}} - \pi_{{Ii,I\ell \left( {LP} \right)}} } \right),$$

9 $$R_{A} \cong \sigma_{u}^{2} \mathop \sum \limits_{{i \in U_{{\text{I}}} }} \mathop \sum \limits_{\ell \ne i} \frac{{N_{i} }}{{\pi_{{{\text{I}}i}} }}\frac{{N_{\ell } }}{{\pi_{{{\text{I}}\ell }} }}\overline{\rho }_{i,\ell } \;\pi_{{{\text{I}}i,{\text{I}}\ell \left( {LP} \right)}}$$

where $\pi_{{{\text{I}}i,{\text{I}}\ell \left( {LP} \right)}}$ is the first-stage joint inclusion probability of clusters $U_{i}$ and $U_{j}$ of the local pivotal sampling design, and $\pi_{{{\text{I}}i,{\text{I}}\ell \left( {FPPS} \right)}}$ is the first-stage joint inclusion probability of a standard PPS sampling design. Since the joint inclusion probabilities $\pi_{{{\text{I}}i,{\text{I}}\ell \left( {FPPS} \right)}} \;{\text{and}}\;\pi_{{{\text{I}}i,{\text{I}}\ell \left( {LP} \right)}}$ are generally unknown, we may estimate them via Monte Carlo simulation. Expressing the spatial correlation as in Eq. (6), the value of $R_{A}$ depends mainly on the size of parameter $\rho$: the closer is $\rho$ to the value 1, the lower the decrease with the distance of the spatial correlation and, hence, the greater the gain in efficiency. Similarly, the terms $C^{*}$ and $R_{C}$ may be approximately defined as:

10 $$C^{*} \cong \mathop \sum \limits_{{i \in U_{{\text{I}}} }} \mathop \sum \limits_{{j \in U_{i} }} \mathop \sum \limits_{k \ne j} \sigma_{u}^{2} \rho_{ij,ik} \frac{1}{{\pi_{{{\text{I}}i}} \pi_{{{\text{II}}i}}^{2} }}\left[ {\frac{{\overline{n}\left( {\overline{n} - 1} \right)}}{{N_{i} \left( {N_{i} - 1} \right)}} - \pi_{{{\text{II}}ij,{\text{II}}ik\quad \left( {LP} \right)}} } \right],$$

11 $$R_{C} \cong \mathop \sum \limits_{{i \in U_{{\text{I}}} }} \mathop \sum \limits_{{j \in U_{i} }} \mathop \sum \limits_{k \ne j} \sigma_{u}^{2} \rho_{ij,ik} \frac{1}{{\pi_{{{\text{I}}i}} }}\frac{{\pi_{{{\text{II}}ij,{\text{II}}ik\quad \left( {LP} \right)}} }}{{\pi_{{{\text{II}}i}}^{2} }},$$

Because the distances between units in the same cluster are tiny, the effectiveness of the second stage derived from local pivotal sampling may be poor. Indeed, the correlation values $\rho_{ij,ik}$ remain uniformly high in this situation. Furthermore, spreading in the second stage may be more difficult due to a lack of professional skills to carry on that exercise. Therefore, a feasible strategy should ensure that the second-stage selection is carried out autonomously in each sample cluster. In this case, it would be better to adopt an SRSWOR design.

Let us now consider the balancing. First, let us note that it is not conceivable to balance directly on the ${\varvec{X}}_{i}$ totals at the cluster level since the variables ${\varvec{x}}_{ij}$ that influence the spread of a pandemic are strictly related to personal behaviour (e.g., number of people met and number of journeys). These variables are usually unavailable in the sampling frames. Individuals’ age, class and sex may represent a good proxy of the unknown ${\varvec{x}}_{ij}$ values. A rational strategy is to identify a vector ${\varvec{z}}_{i}$ of known auxiliary variables at the cluster level, which we can assume to be correlated to the dissemination of the pandemic. We can then define a working model:

12 $$y_{ij} = \tilde{y}_{\left( z \right)i} + u_{\left( z \right)ij}$$

where $\tilde{y}_{\left( z \right)i} = h\left( {{\varvec{z}}_{i} } \right),$ denotes a Lipschitz continuous function, which returns the same value for all individuals in the same cluster, and the residual $u_{\left( z \right)ij}$ is given by $u_{\left( z \right)ij} = u_{ij} + g\left( {{\varvec{x}}_{ij} } \right) - h\left( {{\varvec{z}}_{i} } \right).$

We may express the component $B^{*}$ as:

13 $$B^{*} = V_{P} \left[ {\mathop \sum \limits_{{i \in S_{{\text{I}}} }} N_{i} h\left( {{\varvec{z}}_{i} } \right)\frac{1}{{\pi_{{{\text{I}}i}} }} - \mathop \sum \limits_{{i \in U_{{\text{I}}} }} N_{i} h\left( {{\varvec{z}}_{i} } \right)} \right],$$

where $B^{*}$ is the sampling variance of the predictions $N_{i} h\left( {{\varvec{z}}_{i} } \right)$ of the totals $Y_{i}$ obtained by Model (12), whereas $B$ is the sampling variance of the theoretical values $\tilde{Y}_{i}$ derived by Model (2) when generating the data.

In the context of the pandemic, possible choices of ${\varvec{z}}_{i}$ are as follows:

14 $${\varvec{z}}_{i} = {\overline{\mathcal{V}}}_{i},\quad {\varvec{z}}_{i} = {\varvec{k}}_{i} ,\quad {\varvec{z}}_{i} = \left( {{\overline{\mathcal{V}}}_{i} ,\quad {\varvec{k}}_{i}^{\prime } } \right)^{\prime } ,$$

where ${\overline{\mathcal{V}}}_{i} = {\mathcal{V}}_{i} /N_{i}$ and ${\varvec{k}}_{i}$ is the vector of the geographical coordinates of cluster $U_{i}$.

The first equation in (14) assumes that being infected depends mainly on the average number of verified infected people in the cluster. In contrast, the second equation in (14) implies that the probabilities of being infected are a function of the geographical coordinates of $U_{i}$ only. Finally, the third equation in (14) identifies both the average number of verified infections and the geographical coordinates of $U_{i}$ as possible influencing factors on the probability of being infected. Under Model (12), we have $D^{*} = 0$ and $R_{D} = D.$

Finally, we note that balancing the sample requires the availability of a population register with balancing variables ${\varvec{x}}_{ij}$ available for every individual. However, this is not what happens in most situations we may encounter.

To summarize the results presented in this section, the best strategy to follow in practical cases is to balance the first-stage sampling on the $N_{i} h\left( {{\varvec{z}}_{i} } \right)$ variables to guarantee that the term $B^{*}$ is nullified as a consequence of the fact that $\mathop \sum \limits_{{i \in S_{{\text{I}}} }} N_{i} h\left( {{\varvec{z}}_{i} } \right)\frac{1}{{\pi_{{{\text{I}}i}} }} \cong \mathop \sum \limits_{{i \in U_{{\text{I}}} }} N_{i} h\left( {{\varvec{z}}_{i} } \right)$. Moreover, we can spread the first-stage sampling and have a small $\pi_{{{\text{I}}i,{\text{I}}\ell }}$ when $\overline{\rho }_{i,\ell }$ is large. Indeed, in this case, the dominant term of $AV\left( {\hat{Y}} \right)$ is:

15 $$AV\left( {\hat{Y}} \right) \cong R_{A} + R_{B} + C + D + E - V_{M} \left( Y \right)$$

This strategy approaches the theoretical optimum $E - V_{M} \left( Y \right)$ if the terms $R_{A} \;{\text{and}}\;R_{B}$ are small and the components $C\;{\text{and}}\;D$ (derived from the second-stage sampling) are comparatively lower than the terms $A\;{\text{and}}\;B$ derived from the first-stage sampling. Furthermore, we may introduce two indicators of efficiency. The first is the feasible sampling design's efficiency index ($eff$) defined as:

16 $$eff = 100\frac{{R_{A} + R_{B} + C + D + E}}{A + B + C + D + E}.$$

In addition, the difference between above design's efficiency and the maximum efficiency ($eff_{max}$) can be computed as

17 $$eff_{max} = 100\frac{E}{A + B + C + D + E}.$$

## Simulation results

### Simulation of pandemic spread

In this section, we evaluate the performance of our proposed sampling methods using a simulated dataset representing an artificial population. The algorithm used for generating the data are extensively described in Alleva et al. (2022). The R package *episampler* (Nardelli 2020), containing all the codes to generate the dataset, is freely available online. Alleva et al. (2022) adopted an augmented SIR model that best represents the characteristics of the SARS-CoV-2 epidemic. In fact, in the original formulation (Kermack and McKendrick 1927), the authors considered the infected people divided into only two categories, namely, ‘verified’ (identified by a positive swab from health screening) and ‘unverified’, i.e., those who were not aware of being infected. This model has also been used extensively to analyse the evolution of the COVID-19 pandemic (see among others Taimoor et al. 2022). In contrast, in our formulation, we also distinguish infected individuals with or without symptoms. In fact, regarding the transmission chain, the ‘unverified’ infected are not isolated in quarantine and continue to move and meet other people, thus increasing the spread of the epidemic. Furthermore, in contrast to the original formulation, those that are removed from the list of susceptibles are further distinguished in the categories of « healed» or « dead».

We considered an artificial population of individuals distributed across 400 spatial units laid on a regular 20-by-20 square lattice grid. The structure of the map is intentionally generic: it can represent both a city divided into neighbourhoods or a small region divided into several administrative areas. The density of the population residing in each cell was generated considering different spatial distributions. In three different experiments, we generated 20,000 individuals distributed with a spatial autocorrelation parameter equal to 0.3, 0.5 or 0.7 to reproduce different patterns of spatial agglomeration in urban settlements (Xu et al. 2010). However, in what follows, we report only the results characterized by an autocorrelation parameter equal to 0.5 because the other two cases do not present significant differences.

**Fig. 1:**
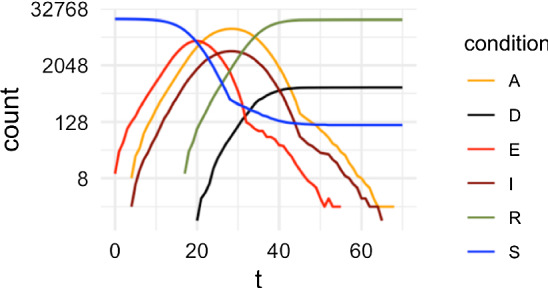
Epidemic curves for the generated map

The movements of the individuals were simulated as follows: each day, some of the individuals go to the four central cells considered the points of attraction (e.g., the city centre for work or leisure). Contagion is simulated to occur during the meeting of individuals and during their movements in the geographical space with a probabilistic mechanism. Epidemic curves are then simulated with the mobility and social interaction mechanism and divided into two phases. In the first 4 weeks (Phase 1), the interaction corresponds to a situation of normality, while in the following 6 weeks (Phase 2), we simulated a state of lockdown. The main results are reported in Fig. 1, which displays the time trend of 6 categories: the susceptible (S), those exposed to the virus (E), those infected with symptoms (I) and without symptoms (A), and those removed from the population either because they are healed (R) or dead (D). The trend of the various categories of the model closely resembles those observed in many empirical situations, e.g., those of the first wave observed in the different Italian regions affected by the SARS CoV-2 pandemic from February to June 2020.

**Fig. 2:**
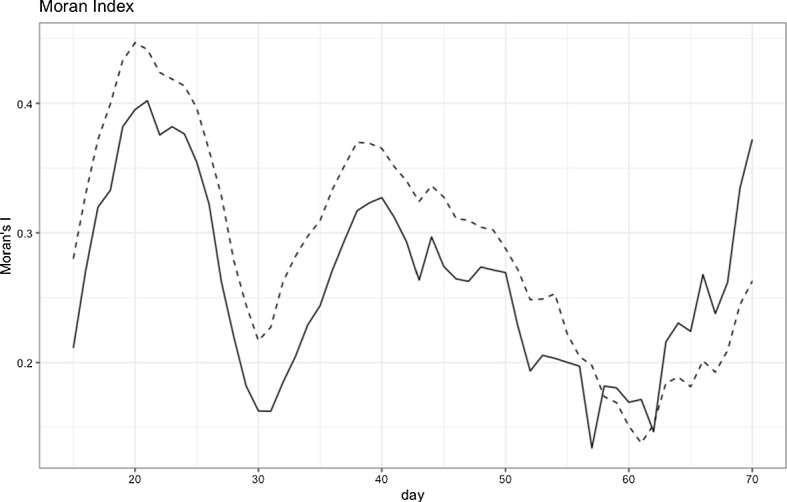
Evolution of Moran’s spatial correlation coefficient over time for known infected (I) (dashed line) and total infected (I + A) (solid line) populations in simulation

To measure the impact of the spatial correlation of the susceptible population and mobility, for each day of the pandemic, we calculated the Moran index (Moran 1950) for both the known infected and the total infected (I + A) populations. Figure 2 shows that the spatial correlation of the infected is more attributable to the mechanisms of mobility and social interactions than to the geographical distribution of the population because it changes dramatically over time. Moreover, considering the distribution of the known infected (I) and of the total infected (A + I), the spatial correlation of the two variables follows the same pattern in the homogeneous screening simulation. We can observe that, quite intuitively, the spatial correlation increases in the early phase when the outbreaks are still limited. Conversely, when the epidemic spreads throughout the map, the spatial correlation declines to a minimum, which is reached at the epidemic peak. Once the infected curve reached the plateau (Day 29), in areas with lower incidence, the total number of infected people decreased faster thanks to lockdown policies, resulting in a subsequent new increase in spatial correlation.

### Evaluation of different sampling designs

For comparison, our experiment considers six possible first-stage alternative sampling designs as follows: (1) fixed-size probability proportional to size (FPPS), (2) local pivotal (LP) method, (3) local cube method (LC) based on verified infected (LCBV) in which the balancing variables use the first equation in (14) to specify the ${\varvec{z}}$ variables, (4) LC method based on geography (LCBG) in which the balancing uses the second specification of (14), (5) LC method based on both the verified infections and on geography (LCBVG) where the balancing is based on the third specification of (14), and (6) LC method based on the variables influencing the pandemic at the unit level (LCBI).

Given the way we created the pandemic simulation, the variables influencing the probability of becoming infected at the level of a single unit of the design LCBI are the following: number of people, total distance and number of travels, total number of contacts and rate of the known infected of the cluster. The designs FPPS and LCBI represent the lower and upper bounds of efficiency, respectively. The LCBI design is not feasible in practice since we would have to record all relevant auxiliary information at the unit level that influenced the spread of the infection in the sampling frame. Using the artificial population generated as described in Sect. 5.1, we simulated a sample survey at three moments of time: Day 15 (during the ascending phase of the epidemic), Day 29 (at the peak) and Day 43 (during the descending phase in the lockdown period). For each combination of the simulation parameters, we repeated the Monte Carlo exercise for 50,000 runs to ensure the convergence of the results for different sample cells (20, 40, 80 and 160 out of 400) with different numbers of people sampled in each cell (1, 3, 5 and 7) on different days (Days 15, 29 and 43 of the pandemic). For simplicity, in Table 1, we report the true value, estimated value obtained as the mean of the various simulation runs, relative bias expressed in absolute terms (RAB) and standard error (SE) of the estimates for Day 15 with 3 people for 80 cells. The results of the simulations obtained using other parameter combinations do not add further insight. As expected, all sampling methods considered produce unbiased estimates. Furthermore, they provide evidence of consistency, although with very different convergence speeds and significantly lower standard errors than those produced by FPPS sampling. The LCBI displays the lowest standard error. As discussed above, since it would need to capture any pertinent auxiliary data at the individual level, this design is not viable in practice. The LCBV technique exhibits the smallest standard error among the remaining feasible designs.

**Table 1:** Simulation results for Day 15 (3 people–80 cells)

Sampling method	True value	Estimate	RAB	SE
FPPS	1,035	1,048	0.0125	0.37
LP	1,035	1,035	0.0001	0.34
LCBV	1,035	1,034	0.0006	0.25
LCBG	1,035	1,037	0.0015	0.34
LCBVG	1,035	1,028	0.0071	0.32
LCBI	1,035	1,034	0.0006	0.23

To add further insights, in Table 2, we report the standard errors calculated for the three different epidemics. Again, LCBV sampling outperforms the other feasible methods. These results agree with those obtained in previous simulation studies (Grafström et al. 2013).

**Table 2:** Standard error of simulation on different epidemic days (3 people–80 cells)

Sampling method	Day 15	Day 29	Day 43
FPPS	0.37	0.72	0.50
LP	0.34	0.68	0.47
LCBV	0.25	0.58	0.42
LCBG	0.34	0.68	0.47
LCBVG	0.32	0.67	0.46
LCBI	0.23	0.57	0.41

The LCBV method performs best with respect to the other sampling strategies because of the assumption of homogeneous screening. In the previous simulation, we assumed that all the infected people have the same probability of being discovered. Although sometimes empirically grounded (Nishura et al. 2020), this assumption may sometimes be too strong and may not properly represent what occurs.

For this reason, in a further Monte Carlo study, we relax the hypothesis of homogeneity, and we assume the presence of heterogeneity by dividing the map into 4 squared macroregions characterized by different abilities to find infected individuals in each area. For brevity, we omit all results similar to those obtained in the previous simulation. The main difference under the heterogeneity assumption is that the method which is balanced both with space and with known infection (LCBVG) appears to be more robust and it performs better than the one balanced only for the number of known infected people neglecting space. Considering that in practical instances the relationship between known and unknown infected cannot be known a priori, the LCBG may be a good choice. Indeed, it approaches the optimal accuracy provided by the LCBVG sampling.

### Evaluation of various addenda of anticipated variance

To complete our analysis^1^ and refer to the theoretical results derived in Sect. 3, Table 3 reports an evaluation of the size of the terms $A,B,C,D$ and $E$ in Eq. (5) (expressed as a percentage) for Days 7, 15 and 43 of our simulation. We estimated the term $\rho$ of the covariance $\rho_{ij,ik} = \rho^{{\delta_{ij,\ell k} }}$ with the methods of moments.

**Table 3:** Percent value of each component over sum of components $A,B,C,D\;{\text{and}}\;E$

Day	$\rho$	$A$	$B$	$C$	$D$	$E$
7	0.598	59.32	23.73	11.32	0.03	5.60
15	0.635	67.34	17.24	10.31	0.01	5.10
43	0.961	82.10	16.40	1.00	0.01	0.49

Table 4 provides the percentage of the unavoidable first-stage components $R_{A} \;{\text{and}}\;R_{B}$ over the respective terms $A$ and $B$. We compute the term $R_{B}$ by balancing the first stage on the aggregated number of verified infections. The table shows the feasible sampling design's efficiency index ($eff$) and the difference between this design's efficiency and the maximum efficiency ($eff_{max}$).

**Table 4:** Percent ratio of unavoidable first-stage components $R_{A} \;{\text{and}}\;R_{B}$ over respective terms $A\;{\text{and}}\;B$ and indices of efficiency

Day	$\rho$	$\left( {R_{A} /A} \right)100$	$\left( {R_{B} /B} \right)100$^*^	$eff$	$eff - eff_{\max }$
7	0.598	10.3	19.45	27,7	22,0
15	0.635	8.8	15.31	24,0	18,8
43	0.961	0.4	15.72	4,4	3,9

*We compute $R_{B}$ by balancing first stage on aggregated number of verified infected

Looking at the two tables, we can draw the following conclusions: (1) The value of $\rho$, as defined in Formula (4), is relatively high each day and consistently higher than 0.59. (2) The term $A$ is dominant since it represents more than 60% of the sum of the positive components of $AV$. Its importance dramatically increases when the parameter $\rho$ approaches its theoretical maximum, that is, in highly positive spatially correlated situations. (3) The term $B$ represents a relevant component with a relative percent size ranging from 15 to 20. Balancing in the first stage, therefore, turns out to be a good way to reduce sample variability without needing access to individual data. (4) The first-stage components $A$ and $B$ together represent more than 80% of the AV. (5) The second stage-components $C,D$ and $E$ represent a negligible part of the AV being together consistently lower than 20%. The second stage achieves only 20 percent efficiency because most of the variability is observed in the first stage among the different PSUs, while the variability among individuals living nearby (second stage) is much lower. Therefore, using more complex methods that also consider spatial correlation in the second stage can only negligibly improve the efficiency of the method. (6) The local pivotal method can cancel more than 90% of component *A.* Similarly, balancing the first stage on the aggregated number of verified infected eliminates more than 30% of the term *B*.

To summarize, the proposed strategy based on spatially balanced sampling in the first stage and simple random sampling in the second stage achieves a very high level of efficiency since it cancels out more than 70% of the positive components of the AV. The second-stage sampling could achieve only a 20% additional efficiency, but this gain may jeopardize the survey’s feasibility.

## Conclusions and research priorities

The aim of this paper was to improve the current practice in epidemic data collection by introducing sampling designs that exploit the intrinsic peculiarity of data of being positively spatially correlated. In this context, we studied the feasibility and efficiency of two-stage sampling designs to estimate critical parameters of COVID-19 infection. The National Statistical Institutes could implement them rather quickly to provide timely information on the development of the pandemic. We propose to adopt spatially balanced sampling in the first stage and simple random selection in the second stage. This strategy is efficient and feasible. Since the phenomenon of infection is positively correlated, spatial sampling allows gaining efficiency by spreading the first-stage sample over space. The balancing strategy leverages aggregated data on the number of verified infected people often available at the primary unit stage level in many countries. Simple random sampling in the second stage does not require information at the unit level. Since the essential auxiliary variables are only represented at the aggregate level, the strategy we provide is also significant from the perspective of its viability in terms of privacy compliance.

Thanks to a simulation study, the theoretical optimality properties of the estimators were confirmed, and the advantages derived from the introduction of the spatial dimension appear to be highly relevant.

The results obtained in this paper encourage us to extend our research in several directions. Indeed, some developments represent a natural extension of the present proposal. The simulation could be extended by adding information such as age, sex and professional condition that are useful to balance the sample, thus further improving the efficiency of the estimators. Different forms of map structure, population density and mobility schemes could also be introduced to represent different types of urban contexts or regional settlements to tailor the design to different real cases. Other possible developments may concern the adaptation of the proposed method for the selection of sample units to which diagnostic tests seeking to trace the diffusion of the virus can be administered. One good example is the tracing of the variants of COVID-19 observed in the 2021 waves, with a specific focus on their geographical spread.

## Footnotes

1 The code to reproduce the analysis is available on https://github.com/vincnardelli/sampling-efficiency-feasibility.
